# The Effect of Histological CD20-Positive B Cell Infiltration in Acute Cellular Rejection on Kidney Transplant Allograft Survival

**DOI:** 10.1155/2016/7473239

**Published:** 2016-12-12

**Authors:** Yan Jiang, Rending Wang, Huiping Wang, Hongfeng Huang, Wenhan Peng, Wenxian Qiu, Jingyi Zhou, Jianghua Chen

**Affiliations:** ^1^Kidney Disease Center, The First Affiliated Hospital, College of Medicine, Zhejiang University, Hangzhou, China; ^2^Key Laboratory of Kidney Disease Prevention and Control Technology, Zhejiang Province, China; ^3^The Third Grade Laboratory under the National State, Administration of Traditional Chinese Medicine, Hangzhou 310003, China

## Abstract

*Background*. It is controversial whether lymphocyte infiltration exhibited in biopsy specimens is associated with transplant outcomes. This study focused on the effect of CD20-positive B cell infiltration in biopsy specimens from allografts with acute cellular rejection (ACR) in a Chinese population.* Methods*. Altogether, 216 patients transplanted from Sep. 2001 to Dec. 2014 with biopsy-proved ACR (Banff I or Banff II) were included in the analysis. Biopsies were immunostained for CD20 and C4d. Baseline information, serum creatinine and GFR before and after treatment, steroid resistance, response to treatment, graft loss, and survival were analyzed.* Results*. Eighty-three patients were classified into CD20-negative group, and 133 patients were classified into CD20-positive group. Significantly more CD20-negative patients (49/83, 59.0%) received steroid plus antibody therapy compared with the CD20-positive group (52/133, 39.1%) (*P* = 0.004). The response to treatment for ACR did not differ between these two groups. The CD20-positive group had less graft loss (18.8% versus 32.5%, *P* = 0.022) and a better graft survival rate. Further exploration of the infiltration degree suggested that it tended to be positively related to graft survival, but this did not reach statistical significance.* Conclusion*. CD20-positive B cell infiltration in renal allograft biopsies with ACR is associated with less steroid resistance and better graft survival. The presence of CD20-positive B cells is protective for renal allografts.

## 1. Introduction 

Acute rejection (AR) is a major risk factor for chronic allograft nephropathy and renal allograft failure after kidney transplantation [1]. The Banff classification criteria divided AR into acute cellular rejection (ACR) and acute humoral rejection [2]. ACR is also known as “T cell mediated rejection,” as it is associated with cytotoxic T cell infiltration. However, long-term allograft survival has not been completely improved by controlling factors that affect the T cell pathway [3–5]. In addition to T cells, other inflammatory cells, including CD20-positive B cells [6–8], plasma cells [9–11], macrophages [12–15], eosinophils [16–18], and NK cells [19], can also infiltrate grafts in ACR and may affect the severity of rejection and the therapeutic response.

The role of CD20-positive B lymphocytes in renal allograft biopsy specimens is still controversial. Using DNA microarrays and immunohistochemical staining, Sarwal et al. reported the presence of CD20-positive B lymphocytes in the graft interstitium of pediatric recipients with ACR for the first time, and they concluded that it was strongly associated with clinical glucocorticoid resistance and graft loss [7]. Subsequently, Hippen et al. classified ACR biopsies into the CD20-positive group if they demonstrated strong and diffuse staining characteristics, while trace or rare CD20-positivity was recognized as the CD20-negative group [6]. Their research suggested that CD20-positive infiltrates correlated with worse clinical outcomes. Other studies also suggested a correlation between CD20 graft infiltration and steroid-resistant rejection [20, 21]. However, relevant studies have argued that CD20-positive B cells exhibited in biopsies have no effect on clinical outcome [22, 23]. Clatworthy et al. conducted a clinical trial comparing rituximab (an anti-CD20 monoclonal antibody) with daclizumab (an anti-CD25 monoclonal antibody) as induction therapy in nonsensitive kidney transplant recipients, but this trial was suspended because of an excess incidence of ACR in the rituximab group [24]. The authors surmised that this anti-CD20 monoclonal antibody might have cleared immunoregulatory B cells, including B cells in the allograft tissue, which led to a marked increase in ACR. Disagreements that exist in published studies may be because of a relatively small sample size and lack of a unified standard for the definitions of what is CD20-positive and CD20-negative.

The aim of this study was to determine the effects of CD20-positive B cell graft infiltration during ACR on allograft outcome in a Chinese population.

## 2. Patients and Methods

### 2.1. Patients

This is a retrospective study of patients who underwent kidney transplantation between September 2001 and December 2014 at the Kidney Disease Center of the First Affiliated Hospital of Zhejiang University (Hangzhou, China). This study was approved by the Committee of Ethics in Biomedical Research of Zhejiang University. Pathological records, clinical data, test results, and follow-up information of all patients were collected from the electronic medical record system and the kidney transplantation database of our center.

Altogether, 217 patients were identified with biopsy-proven ACR (grade I or II) according to the Banff 2005 criteria and were negative for C4d staining. Excluding one patient who was lost of follow-up after antirejection treatment, 216 patients were included in this analysis. All patients were followed up until June 30, 2015. According to the presence of CD20-positive B cell infiltration, 83 recipients were classified into the CD20-negative group, and 133 were classified into the CD20-positive group. The pathologic types of ACR in these two groups were listed in Table 1.

Most of the recipients received calcineurin inhibitor (CNI) (cyclosporine or tacrolimus) in combination with mycophenolate mofetil (MMF) and steroids as maintenance immunosuppressive regimen, while some received rapamycin in place of CNI. Cyclosporine (CsA) was initiated at 5 mg/kg/d and tacrolimus (FK506) at 0.10–0.15 mg/kg/d. The drug dosage was adjusted according to the plasma concentration. The target plasma concentration for CsA and FK506 was 250–300 *μ*g/L and 8–12 *μ*g/L, respectively, during the first month after transplantation; 200–250 *μ*g/L and 6–10 *μ*g/L, respectively, from the second to the third month; 150–200 *μ*g/L and 4–8 *μ*g/L, respectively, from the fourth to the sixth month; and around 150 *μ*g/L and 3–6 *μ*g/L, respectively, after the sixth month. MMF was started at 1.5–2 g/d for half a month and maintained at 1 g/d thereafter. Methylprednisolone was given at 6 mg/kg on the third postoperative day. Since then, starting from 80 mg/d, with a daily reduction of 10 mg, prednisone was maintained at 10–15 mg/d.

Once acute rejection was proven by allograft biopsy, intravenous methylprednisolone was administrated at 6–10 mg/kg daily for 3 days as pulse therapy. If the serum creatinine (Cr) level decreased more than 50% or went back to the baseline level within 1-2 weeks, the treatment was considered effective. If not, ACR was further treated with OKT3 at 5–10 mg/d or ATG at 100–200 mg/d for 5–7 days. Response to therapy was determined by comparing the serum Cr level measured two weeks after completion of the antirejection treatment to the baseline serum Cr level measured before rejection [25]. Response was considered complete if a decrease in serum Cr level was maximally 125% of baseline and partial if the Cr was 125%–175% of baseline, and it is no-response if Cr was still more than 175% of the baseline or graft loss (back to dialysis or nephrectomy).

### 2.2. Allograft Biopsy and Histopathology

Percutaneous ultrasound-guided renal biopsy was performed in recipients experiencing allograft dysfunction. Kidney allograft pathology diagnosis was made by an experienced renal pathologist (W. H.) according to the Banff 2005 criteria. We only included ACR grade I and grade II. ACR grade III and antibody-mediated rejection were excluded. Vascular rejection refers to the existence of intimal arteritis in the ACR sample, that is, grades IIA, IIB, IIA + IA, IIA + IB, IIB + IA, and IIB + IB, according to the Banff 2005 criteria.

**Figure 1:**
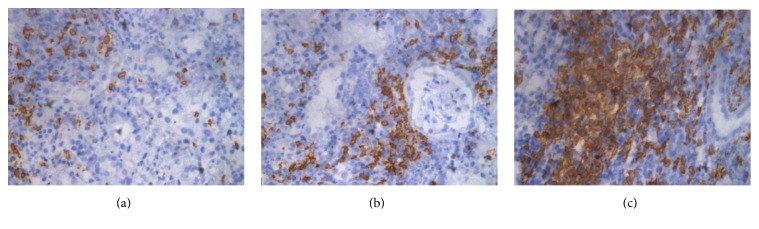
Representative pictures of CD20 immunostaining in ACR (×400): (a) CD20 mild-positive; (b) CD20 moderate-positive; (c) CD20 severe-positive.

Immunohistochemical staining for CD20 and C4d was routinely performed on paraffin sections using the CD20 monoclonal antibody from Zhongshan (Cat number ZA0549, Beijing, China) and the C4d polyclonal antibody from Abcam (Cat number ab36075, Cambridge, UK). Using the same definition as those of Hippen et al., CD20-positive was defined as strong and diffuse staining characteristics, while trace or rare CD20-positivity was assigned to CD20-negative [6]. The CD20-positive specimens were further assessed independently by two authors (W. R. and W. H.) and defined as mild-positive if CD20-positive cells accounted for less than 25% of the inflammatory cells, moderate-positive if CD20-positive cells were 26%–49% of the inflammatory cells, and severe-positive if CD20-positive cells were more than 50% of the inflammatory cells. Representative images are shown in Figure 1. All the patients included in this study were negative for C4d staining.

### 2.3. Statistical Analysis

Continuous variables are expressed as mean ± standard deviation (SD) or median (range). Categorical variables are presented as numbers (frequencies). Normally distributed continuous variables were analyzed using student's *t*-test or one-way ANOVA, and nonnormally distributed continuous variables were analyzed using Mann–Whitney test. Chi-square test was used for categorical variables. Graft/patient survival was analyzed with Kaplan-Meier method and differences between survival curves were calculated by the log-rank test. Factors associated with graft loss on the univariate analysis with *P* < 0.1 were included into a Cox multivariate analysis. All statistical calculations used SPSS 19.0. Two sided *P* < 0.05 was considered as significant difference.

## 3. Results 

### 3.1. Baseline Characteristics

The baseline characteristics of the patients were listed in Table 2. Eighty-three patients were assigned to the CD20-negative group, and 133 patients were classified as CD20-positive. No significant differences in age, gender, cold/warm ischemia time, donor type, primary disease, induction regimen, and prerejection immunosuppressive drugs were observed between these two groups. ACR was diagnosed earlier after kidney transplantation in the CD20-negative group compared with CD20-positive group (median time to ACR, 29 days versus 142 days, *P* = 0.016). As presented in Table 1, the pathologic types differed in two groups (*P* = 0.002). There was significantly more vascular rejection (IIA, IIB, IIA + IA, IIA + IB, IIB + IA, and IIB + IB) in the CD20-negative group (50/83 patients, 60.2%), compared with the CD20-positive group (55/133 patients, 41.4%) (*P* = 0.005).

### 3.2. Antirejection Therapy

In general, CD20-negative patients presented with higher prerejection serum Cr levels, compared with the CD20-positive group (180.1 ± 128.4 versus 130.6 ± 68.1 *μ*mol/L, *P* = 0.002). The same results were obtained with peak Cr at rejection (352.7 ± 242.3 versus 274.1 ± 265.6 *μ*mol/L, *P* = 0.027). No significant differences were observed at any other time point during follow-up (Table 3). Corresponding to this, worse GFR was observed in the CD20-negative group before rejection (47.2 ± 21.3 versus 60.4 ± 21.6 mL/min, *P* < 0.001), and the same results were obtained at the time of rejection (25.0 ± 15.0 versus 30.6 ± 13.3 mL/min, *P* = 0.005). No significant differences between CD20-positive and CD20-negative groups were observed at any other time point during follow-up (Table 4).

Patients in the CD20-positive and CD20-negative groups received similar maintenance immunosuppressive regimen after rejection (Table 5). After ACR, significantly more CD20-negative patients (49/83, 59.0%) received steroid plus antibody therapy (defined as steroid-resistant rejection) compared with the CD20-positive group (52/133, 39.1%) (*P* = 0.004). The response to treatment for ACR did not differ between these two groups.

### 3.3. CD20 Staining and Patient/Graft Survival Rates

**Figure 2:**
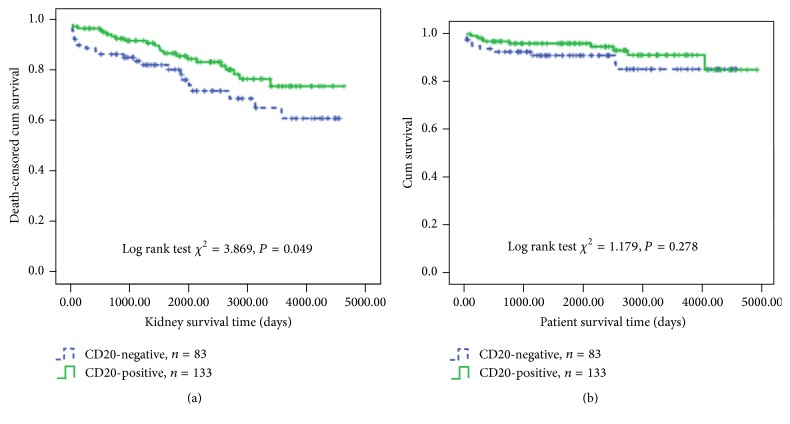
Effects of CD20 staining on (a) death-censored renal allograft survival; (b) patient survival.

More patients in the CD20-negative group (27/83, 32.5%) experienced graft loss compared with the CD20-positive group (25/133, 18.8%), which reached a significant difference (*P* = 0.022).  Figure 2(a) displayed the graft survival over time analyzed by the Kaplan-Meier death-censored method for CD20-positive and CD20-negative groups. CD20-positive infiltration was associated with significantly better allograft survival (*P* = 0.049). There was no significant difference in the patient survival rate between these two groups.

### 3.4. Association of the Degree of CD20 Infiltration and Patient/Graft Survival

**Figure 3:**
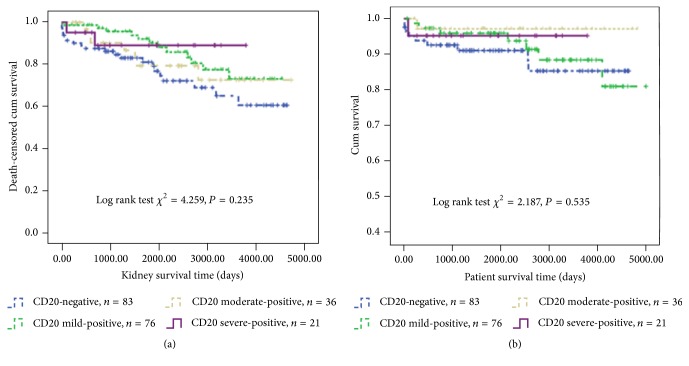
Effects of CD 20 infiltration degrees on (a) death-censored renal allograft survival; (b) patient survival.

We further divided the CD20-positive group into CD20 mild-positive subgroup (*n* = 76), CD20 moderate-positive subgroup (*n* = 36), and CD20 severe-positive subgroup (*n* = 31) according to the percentage of CD20-positive B cells found in the inflammatory cell population.  Figure 3(a) showed that the CD20 severe-positive subgroup tended to have better graft survival compared to the other three groups, but this difference was not significant. Patient survival was similar among these four groups (Figure 3(b)).

### 3.5. Predictor of Graft Loss in a Cox Proportional-Hazards Model

Univariate analysis showed that the CD20-positive infiltration, prerejection immunosuppressive regimen, antirejection therapy, and antirejection response were the factors influencing renal allograft loss. Further multivariate Cox regression analysis revealed that CD20 infiltration was a protective factor for graft loss. Antirejection therapy is another independent risk factor. The adjusted risk ratio of graft loss for steroid plus antibody treatment was 2.316 compared with steroid alone. Compared with the complete response, the adjusted risk ratio of graft loss was 2.538 for partial-response and 13.847 for no-response, as exhibited in Table 6. The prerejection immunosuppressive regimen, which was significant in the univariate analysis, did not reach significance in the multivariate analysis.

## 4. Discussion 

Our study demonstrated that CD20-positive infiltration in the biopsy specimens from the allografts with ACR was associated with less steroid-resistant rejection and better allograft survival. Further exploration of the infiltration degree suggested that it tended to be positively related with graft survival, but without statistical significance. Multivariate Cox regression revealed that CD20-positive infiltration, antirejection therapy, and antirejection response were independent predictors of graft loss. The presence of CD20-positive B cells was a protective factor for graft loss.

B cells are very common in solid organ transplantation. B cells and plasma cells in pathological tissues have been considered as nonspecific effector cells in the past [26, 27]. In 2003, Sarwal et al. first demonstrated the presence of CD20-positive B cells in the graft interstitium of pediatric transplant recipients experiencing ACR [7]. Their study suggested that CD20-positive infiltration was associated with steroid-resistant rejection and worse graft survival. Since then, the role of CD20-positive B cells in ACR has attracted more attention. In adult kidney transplantation, 22% of acute rejection (IA, IB) specimens had CD20-positive infiltration, and it was associated with worse outcome [6]. A mouse model of acute cardiac allograft rejection demonstrated that recipients' B cells participate in indirect alloantigen presentation and play an important role in the progression of acute vascular rejection [28]. Studies analyzing the CD20-positive B cells' infiltration pattern in adult kidney transplantation reported higher serum Cr levels in recipients with clusters of B cells [29]. Some studies have suggested that once recruited into injured grafts, B cells can act as antigen-presenting cells to promote T cell mediated rejection, which is resistant to conventional steroid therapy [30].

Different result was observed, suggesting that the early infiltration of B cells can be beneficial [23, 31]. Scheepstra et al. performed immunostaining for CD20 on 54 biopsy-proven ACR samples, and no correlation was found between the number of CD20 cells, in clusters or in a scattered pattern, and clinical outcome [23]. Increased peripheral blood B cells were detected in the steady recipients [32], instead of those experiencing rejection. CD20 transcription was found to be increased in tolerant kidney transplant recipients [33–35]. Kayler et al. suggested that B cells were not indicators of graft loss or steroid resistance in their analysis of 120 ACR biopsies [36]. This result was proved by other studies [37].

Thus, the role of CD20-positive B lymphocytes in acute cellular rejection is controversial. One of the possible explanations for this is the lack of a unified standard on the definition of CD20-positive and CD20-negative. Sarwal and her colleagues defined CD20-positive as more than 275 CD20-positive cells in a single high power field (HPF) and came to the conclusion that CD20-positive cells were strongly associated with severe graft rejection [7]. Hippen et al. used a qualitative method in their research and defined biopsies with strong and diffuse staining characteristics of CD20-positive cells as the CD20-positive group, while trace or rare CD20-positive cells were recognized as the CD20-negative group [6]. Their research indicated that CD20-positive infiltration was more likely to have steroid-resistant rejection and worse graft survival. In Bagnasco's research, CD20-positive patients (at least one cluster containing more than 100/HPF CD20-positive cells) and CD20-negative patients (the count was below 50/HPF) were compared [37]. No association was found between CD20-positive infiltrates and worse graft outcome using this method. CD20-positive lymphoid clusters, which were defined as any dense cluster of lymphoid cells containing more than 15 CD20-positive B cells by Kayler et al., did not predict steroid resistance or worse graft survival [36].

The definition of CD20-positive and CD20-negative in our study is in line with Hippen et al.'s research. However, our study's findings that CD20-positive infiltration is associated with better clinical outcomes are not consistent with Hippen et al.'s results [6]. Differences exist between our two studies. First, our study included a larger number of ACR biopsies than any published studies so far. A total of 216 cases of biopsy-proven ACR were included in our analysis; 83 samples were assigned to the CD20-negative group, and 133 were classified as CD20-positive. Hippen et al.'s study cohort contained 27 patients and only 6 biopsies were classified as CD20-positive. As mentioned in the discussion by the authors, the relative small sample sizes in their analysis may limit the generalization of their conclusions. Secondly, Hippen et al.'s research was conducted in biopsies with proven Banff IA or IB rejection within the first year after transplantation. Our study included patients with ACR grade I and grade II at any time point after kidney transplantation. The differences in the inclusion criteria of study population may contribute to the different results. Similarly, Scheepstra et al. [23] adopted the same method to assess CD20-positive as mentioned in Sarwal et al.'s work [7], but they reported different results. Thus, besides the lack of a uniformed definition of CD20-positive and CD20-negative, a relatively small sample size, different study populations (pediatrics versus adults; different subgroups of ACR), differences in the induction and maintenance immunosuppression therapy, different follow-up durations, and other factors can contribute to the inconsistency in the studies concerning the role of CD20-positive infiltration in ACR.

The prognostic study of CD20-positive cells in ACR helps to select future treatment, in particular, whether or not to apply B-cell-depleting agents in steroid-resistant rejection. Rituximab, an anti-CD20 monoclonal antibody, was first applied in the treatment of lymphoma. It has been proven effective in treatment of a number of hematological diseases and autoimmune diseases [38–40]. In the aspect of transplantation, rituximab has been used for desensitization of panel reactive antibody, anti-HLA antibody, and anti-ABO antibody before transplantation as induction regimen and treatment of humoral rejection after transplantation [41–47]. In 2002, a cardiac transplant patient with vascular rejection refractory to plasmapheresis was successfully treated with rituximab, which was the first documented application of an anti-CD20 monoclonal antibody in the aspect of rejection [48]. Later, other case reports confirmed that CD20 monoclonal antibody was effective in the treatment of vascular rejection in heart and pancreas transplantation [49–51]. At present, rituximab is applied to treat steroid-resistant rejection after renal transplantation. It can clear DSA and B cells to improve outcomes after renal allograft rejection [52]. After treatment, the reconstruction of peripheral B cells requires 6 to 9 months. The CD20-positive B cells in graft are significantly reduced after treatment, but the studies of the reconstruction of B cells in grafts are rather rare [53]. Our results showed that CD20-positive infiltration in patients with ACR appeared to have a protective effect on graft outcome. Additionally, a clinical trial which planned to use rituximab in nonsensitive kidney transplant recipients was forced to stop because of increases in rejection [24]. Thus, the administration of anti-CD20 monoclonal antibody in patients with ACR requires careful consideration.

## 5. Conclusion

CD20-positive B cell infiltration in renal allograft biopsies with ACR is associated with less steroid-resistant rejection and better graft survival. The presence of CD20-positive B cells is protective for renal allografts.

## Figures and Tables

**Table 1:** Distribution of Banff diagnosis stratified by CD20 staining.

Type (grade)	CD20-negative (*n* = 83)	CD20-positive (*n* = 126)	*P* = 0.002
IA	30	65	
IB	3	13	
IIA	45	34	
IA + IIA	3	13	
IB + IIA	1	5	
IA + IIB	0	2	
IIB	1	1	

**Table 2:** Patient baseline characteristics stratified by CD20 staining.

Characteristics	CD20-negative (*n* = 83)	CD20-positive (*n* = 133)	*P* value
Sex (male/female)	54/29	94/39	0.387
Age at transplantation (years)	39.1 ± 12.0	37.5 ± 11.5	0.332
Warm ischemia time (min)	5.5 ± 1.9	5.5 ± 2.4	0.657
Cold ischemia time (min)	442 ± 204	448 ± 209	0.792
Donor type			0.863
Living	17 (20.5%)	26 (19.5%)	
Deceased	66 (79.5%)	107 (80.1%)	
Primary disease			0.445
Chronic nephritis	75 (90.4%)	119 (89.5%)	
AKPD	6 (7.2%)	5 (3.8%)	
Diabetic nephropathy	0 (0)	2 (1.5%)	
Gouty nephropathy	0 (0)	2 (1.5%)	
Hereditary nephropathy	0 (0)	2 (1.5%)	
Others	2 (2.4%)	3 (2.2%)	
Number of HLA mismatches	3.11 ± 1.22	2.98 ± 1.26	0.468
Induction regimen			0.498
CD25 monoclonal antibody	35 (42.2%)	46 (34.6%)	
ATG/OKT3	4 (4.8%)	9 (6.8%)	
None	44 (53.0%)	78 (58.6%)	
PRA > 10% – number (%)			
After transplant	4 (4.8%)	2 (1.5%)	0.149
At rejection	3 (3.6%)	5 (3.8%)	0.956
Prerejection immunosuppressant arms			0.230
FK506 + MMF +Pred	53 (63.9%)	76 (57.1%)	
CSA + MMF + Pred	27 (32.5%)	53 (39.8%)	
Rapamycin + MMF + Pred	1 (1.2%)	2 (1.5%)	
CSA + rapamycin + Pred	2 (2.4%)	0	
CSA + AZA + Pred	0 (0)	2 (1.5%)	
Median days to ACR (range) (days)	29 (3–3878)	142 (3–3398)	0.016

PRA, panel reactive antibody; AZA, azathioprine; CSA, cyclosporine; FK506, tacrolimus; MMF, mycophenolate mofetil; PRED, prednisone.

**Table 3:** Serum creatinine values during follow-up.

	CD20-negative	CD20-positive	*P* value
Before rejection	180.1 ± 128.4 (83)	130.6 ± 68.1 (133)	0.002
Peak	352.7 ± 242.3 (83)	274.1 ± 265.6 (133)	0.027
After biopsy			
1 month	183.2 ± 143.2 (80)	160.1 ± 171.8 (132)	0.313
3 months	150.6 ± 102.5 (62)	135.8 ± 55.6 (113)	0.293
6 months	127.4 ± 50.9 (63)	142.6 ± 68.7 (125)	0.121
12 months	126.9 ± 43.7 (64)	144.7 ± 97.9 (110)	0.101
24 months	127.1 ± 49.5 (58)	131.0 ± 80.0 (91)	0.742
36 months	114.1 ± 49.5 (38)	129.4 ± 58.2 (69)	0.173
48 months	116.8 ± 77.1 (26)	122.0 ± 55.6 (52)	0.734
60 months	113.0 ± 72.0 (23)	116.4 ± 36.5 (47)	0.788

**Table 4:** GFR values during follow-up.

	CD20-negative	CD20-positive	*P* value
Rerejection	47.2 ± 21.3 (83)	60.4 ± 21.6 (133)	<0.001
Peak	25.0 ± 15.0 (83)	30.6 ± 13.3 (133)	0.005
After Biopsy			
1 month	47.2 ± 21.8 (80)	52.5 ± 19.8 (132)	0.070
3 months	54.0 ± 20.2 (62)	55.6 ± 19.4 (113)	0.641
6 months	58.5 ± 18.1 (63)	54.8 ± 20.6 (125)	0.224
12 months	58.1 ± 19.8 (64)	57.3 ± 22.7 (110)	0.804
24 months	58.2 ± 17.4 (58)	60.6 ± 23.7 (91)	0.502
36 months	66.7 ± 25.0 (38)	59.8 ± 20.5 (69)	0.123
48 months	69.3 ± 24.1 (26)	62.6 ± 21.1 (52)	0.212
60 months	68.3 ± 20.3 (23)	64.1 ± 21.4 (47)	0.428

**Table 5:** Antirejection therapy and response to treatment.

	CD20-negative	CD20-positive	*P* value
	(*n* = 83)	(*n* = 133)	
Postrejection immunosuppressant arms			0.898
FK506 + MMF + Pred	69 (83.1%)	113 (85.0%)	
CSA + MMF + Pred	13 (15.7%)	18 (13.5%)	
Rapamycin + MMF + Pred	1 (1.2%)	2 (1.5%)	
Antirejection therapy			0.004
Steroids	34 (41.0%)	81 (60.9%)	
Steroids + antibody	49 (59.0%)	52 (39.1%)	
Response to treatment			0.232
Complete	55 (66.2%)	86 (64.7%)	
partial	11 (13.3%)	28 (21.0%)	
No response	17 (20.5%)	19 (14.3%)	

**Table 6:** Cox regression hazard ratios for renal allograft failure.

	Univariate			Multivariate		
	RR	95% CI	*P*	RR	95% CI	*P*
CD20-positive infiltrates	0.506	0.293–0.872	0.014	0.570	0.327–0.995	0.048
Prerejection immunosuppressive regimen	0.392	0.221–0.696	0.001	0.621	0.356–1.083	0.093
Antirejection therapy (combination versus MMP)	3.142	1.724–5.728	<0.001	3.316	1.677–5.958	<0.001
Response to treatment			<0.001			<0.001
Partial versus complete response	2.613	1.129–6.048	0.025	2.538	1.078–5.974	0.033
No-response versus complete response	13.410	7.032–25.570	<0.001	13.847	7.018–27.321	<0.001

RR, relative risk; CI, confidence interval.
